# A murine sinonasal infection model recapitulates key features of clinical chronic rhinosinusitis

**DOI:** 10.1099/jmm.0.002152

**Published:** 2026-04-08

**Authors:** Emily J. Vanderpool, Kendra P. Rumbaugh

**Affiliations:** 1 Department of Surgery, Texas Tech University Health Sciences Center, Lubbock, TX, USA

**Keywords:** disease models, mouse infection, *Pseudomonas aeruginosa*, sinusitis, *Staphylococcus aureus*

## Abstract

**Introduction.** Chronic rhinosinusitis is a difficult-to-treat, recurrent inflammatory condition of the nose and paranasal sinuses with global prevalence. Despite its impact on patient quality of life and its cost to the healthcare system, the pathogenesis of chronic rhinosinusitis (CRS) remains poorly understood. Additionally, while the presence of bacteria in CRS has been confirmed by numerous studies, their influence on disease symptoms is unclear. Disease-relevant models can help resolve these questions.

**Hypothesis.** We hypothesized that bacterial inoculation could drive CRS-associated symptoms in a murine model.

**Aim.** To characterize host–microbe interactions in a murine model of sinonasal bacterial infection.

**Methodology.** *Staphylococcus aureus* and/or *Pseudomonas aeruginosa* were inoculated in the nasal cavity of Swiss Webster, C57Bl/6, Balb/c and B6.Cg-*Prkdc*^scid^/SzJ severe combined immunodeficient (SCID) mice. Systemic cytokine response was quantified with a multiplexed enzyme-linked immunosorbent assay, and local histological alterations were quantified using haematoxylin and eosin as well as Alcian Blue–Periodic Acid–Schiff-stained sinonasal sections.

**Results.** Intranasal bacterial inoculation induced symptoms of CRS in murine sinonasal cavities. Dual species inoculation generated a unique response compared to single species. Repeated inoculations did not result in bacterial clearance from immunological priming. While Swiss Webster and C57Bl/6 mice demonstrated the greatest magnitude of responses, Balb/c mice demonstrated a protective response, generally downregulating cytokines and attempting to prevent further tissue damage. SCID mice demonstrated effective clearance of *P. aeruginosa* by innate immunity, but maintenance of *S. aureus*.

**Conclusion.** Pathogenic bacteria are able to persist and drive the development of symptoms associated with clinical CRS in a murine model. Bacterial interactions and host factors influence CRS-associated inflammation. By investigating host responses from a number of mouse genetic backgrounds, the heterogeneity of disease presentation in CRS can be modelled, and strategies for infection management can be evaluated as potential therapeutic targets.

**Impact Statement**

Host–microbe interactions and the role of bacteria in chronic rhinosinusitis (CRS) are not well defined. Disease-relevant models of CRS are needed in order to better understand disease sequela and develop novel therapeutics. Herein, we demonstrate pathogenic sinonasal bacterial infection mimicking clinical CRS in an accessible murine model.

## Introduction

Chronic rhinosinusitis (CRS) is a persistent inflammatory condition affecting the nose and paranasal sinuses, significantly contributing to the global health burden in both children and adults, as well as the overuse of antibiotics [1]. CRS is characterized by at least 12 weeks of facial pain or pressure, sinonasal obstruction, nasal discharge and a diminished or lost sense of smell and taste [2]. In some cases, patients develop nasal polyps, which are inflammatory outgrowths of epithelial tissue [3]. CRS without nasal polyps is commonly associated with type 1 inflammatory responses in the Western population, which are characterized by Th1 response, IFN-*γ* and phagocyte activation [4]. CRS with nasal polyps, by contrast, is linked to type 2 inflammatory outcomes, including Th2 responses such as elevated IL-4, IL-5 and IL-13 [4]. Polyps and the associated immune responses are just one example of the heterogeneity of disease in CRS.

Across different disease presentations, the condition leads to frequent medical visits, substantial healthcare costs and a reduced quality of life, including a documented association with clinical depression [5,7]. Additionally, first-line treatments fail to adequately control symptoms in ~40% of patients, many of whom ultimately require functional endoscopic sinus surgery [8]. Even after surgical intervention, some patients need one or more revision procedures to manage their symptoms [9]. Despite affecting 10–15% of American adults, the underlying causes of CRS remain poorly understood, highlighting the need for further research into disease progression and potential therapeutic approaches [10].

Pathogenic bacteria have been frequently identified in the sinonasal cavity of patients with CRS [11,13]. Biofilms, or protected aggregates of bacterial cells, have also been widely identified in CRS patients, along with significant shifts in sinonasal microbiota composition [11,14, 15]. Notably, patients with confirmed sinonasal biofilms tend to have worse prognoses [16]. These findings may help explain CRS’s resistance to treatment, as pathogenic bacteria may be antibiotic resistant and induce inflammatory responses, as biofilms are inherently difficult for the host immune system to eliminate and as microbial dysbiosis is frequently linked to chronic inflammatory diseases [17,18]. Thus, in a broad sense, bacteria have been hypothesized to influence the persistence and severity of CRS. However, the precise role of bacteria in CRS pathogenesis remains unclear.

To better understand CRS disease mechanisms and the role of bacteria, relevant disease models are essential. Various animal models have been developed, but each has limitations that impact their relevance to human disease and microbiological interpretation, such as allergen-based models and bacterial exoproduct-based models [19,20]. For example, many previous CRS animal models have relied on the use of a surgical sponge to obstruct the sinonasal cavity [21,23]. While this strategy may improve bacterial retention, it is a major deviation from the normal CRS pathophysiology and more accurately represents a foreign body infection model than a CRS infection model. To address these challenges, we aimed to establish an accessible murine model of sinonasal infection capable of effectively evaluating bacterial contributions to disease progression and host immune responses.

In this model, we investigated the intranasal inoculation of two key bacterial pathogens, *Staphylococcus aureus* (SA) and *Pseudomonas aeruginosa* (PA). SA is the most frequently detected bacterial species in CRS patient samples [15,24, 25]. PA is also implicated in CRS and is often associated with acute disease exacerbations [26,27]. For example, a clinical study found that individuals with a history of sinusitis were 11.57 times more likely to be colonized with PA than healthy controls [28]. Both SA and PA possess virulence factors that facilitate immune evasion, adherence to and invasion of host cells, disruption of immune responses and degradation of host tissues. Using these virulence factors, both species have the potential to stimulate symptoms of CRS. By evaluating bacterial retention, host immune responses and sinonasal histopathology in our murine model, we aimed to determine the extent to which these pathogens drive CRS-like disease characteristics.

## Methods

### Bacterial strains and growth conditions

Xen36, a *lux*-expressing derivative of the Wright strain of SA, and luminescent PAO1 (PAO1wLux), a PA strain carrying the plasmid pQF50-*lux*, were used in these experiments [29,30]. Briefly, strains were streaked from cryogenic stocks onto selective media, mannitol salt agar for SA and *Pseudomonas* isolation agar for PA, and incubated at 37 °C for 24 h. Colonies from these plates were then used to inoculate overnight cultures in brain–heart infusion broth, grown with shaking at 37 °C. Subcultures were subsequently prepared from the overnight cultures and incubated with shaking for an additional 3 h.

### Intranasal treatments

*Lipopolysaccharide*: Nasal instillation of 15 µl of 3 mg ml^−1^ lipopolysaccharide (LPS) derived from *Escherichia coli* was utilized to establish a standard of comparison for infected mice vs. uninfected mice [31,33].

### Mouse strains and nasal inoculations

This study was approved by the Institutional Animal Care and Use Committee at Texas Tech University Health Sciences Center. Female Swiss Webster, C57BL/6, Balb/c (Charles River Laboratories) and B6.Cg-*Prkdc*^scid^/SzJ (SCID, Jackson Laboratory) mice, aged 5–8 weeks, were used for these studies. Bacterial inoculations were prepared at a concentration of 10⁸ c.f.u. ml^−1^. For SA+PA co-inoculations, bacterial subcultures were combined in a 1 : 1 ratio immediately before inoculation. Mice were anesthetized with isoflurane, and a total of 10 µl of the bacterial suspension was applied externally to the nares via pipetted drops, which the animals inhaled during respiration. Throughout the experiments, mice were monitored for normal breathing and signs of illness.

### Quantification of c.f.u. from nasal tissue

After euthanasia, the nasal cavities were dissected anterior to the orbit to enable more precise bacterial enumeration compared to lavage [34]. The dissected tissue was homogenized in PBS using 2.4-mm metal beads (Fisher Scientific) and processed with a homogenizer (FastPrep-24, MP Biomedicals) for three 60 s cycles at 5 m s^−1^. The resulting homogenates were serially diluted in PBS, plated on selective media and incubated at 37 °C for 24–48 h. To differentiate the inoculated bacterial strains from normal nasal flora, particularly for SA, lux-expressing strains were imaged using in vivo imaging system (IVIS). Reported c.f.u. values for Xen36 and PAO1wLux were specifically confirmed through IVIS imaging.

### Serum cytokine analysis

Whole blood was collected via exsanguination and allowed to clot at room temperature for 20 min. The samples were then centrifuged at 3,000 r.p.m. for 10 min to separate the serum, which was collected and stored at −80 °C. Cytokine levels were quantified using a multiplex ELISA kit (Mouse Proinflammatory Panel 1, Meso Scale Discovery), measuring TNF-*α*, KC/GRO (CXCL1), IL-6, IL-5, IL-2, IL-1*β*, IL-10, IFN-*γ*, IL-4 and IL-12p70. IL-4 and IL-12p70 levels were below the assay’s detection limit for all groups. Before analysis, serum samples were thawed, and total protein concentration was determined using the Qubit Protein Broad Range kit. Serum samples from an average of three mice per group were run in duplicate or triplicate. ELISA plates were processed using the Meso QuickPlex SQ 120, and cytokine concentrations were calculated based on standard curves generated by the Discovery Workbench software (Meso Scale Discovery) [35].

### Nasal histology and imaging

Skulls with lower mandibles removed were fixed in Carnoy’s solution (Spectrum Chemical) and decalcified in CalRite (Richard-Allan Scientific) for a minimum of 24 h each [21,36, 37]. After decalcification, the nasal cavities were sectioned and stored in 70% ethanol until embedding. Tissue sections were embedded in paraffin following the Small Animal Protocol from the TTUHSC Histology Research Core and then sectioned at 5-µm thickness and either left unstained or stained with haematoxylin and eosin. Subsequently, a subset of slides was stained with Alcian Blue-Periodic Acid-Schiff (Newcomer Supply). Sections from an average of three mice per group were imaged and analysed. Images were captured using a Nikon Eclipse Epi-fluorescent microscope equipped with a Digital Sight 10 camera, with Nikon Elements Software used for acquisition and FIJI for analysis. Goblet cells, nasal-associated lymphoid tissue (NALT) and immune cells above the dorsal meatus were quantified using particle analysis from at least two separate 20× fields of view (FOV) per mouse. Trainable Weka Segmentation was applied to quantify Bowman’s glands, epithelial area and exudate, using two equivalent 4× FOVs per mouse.

## Results

### Bacterial inoculation results in host responses that reflect features of clinical CRS

**Fig. 1.**
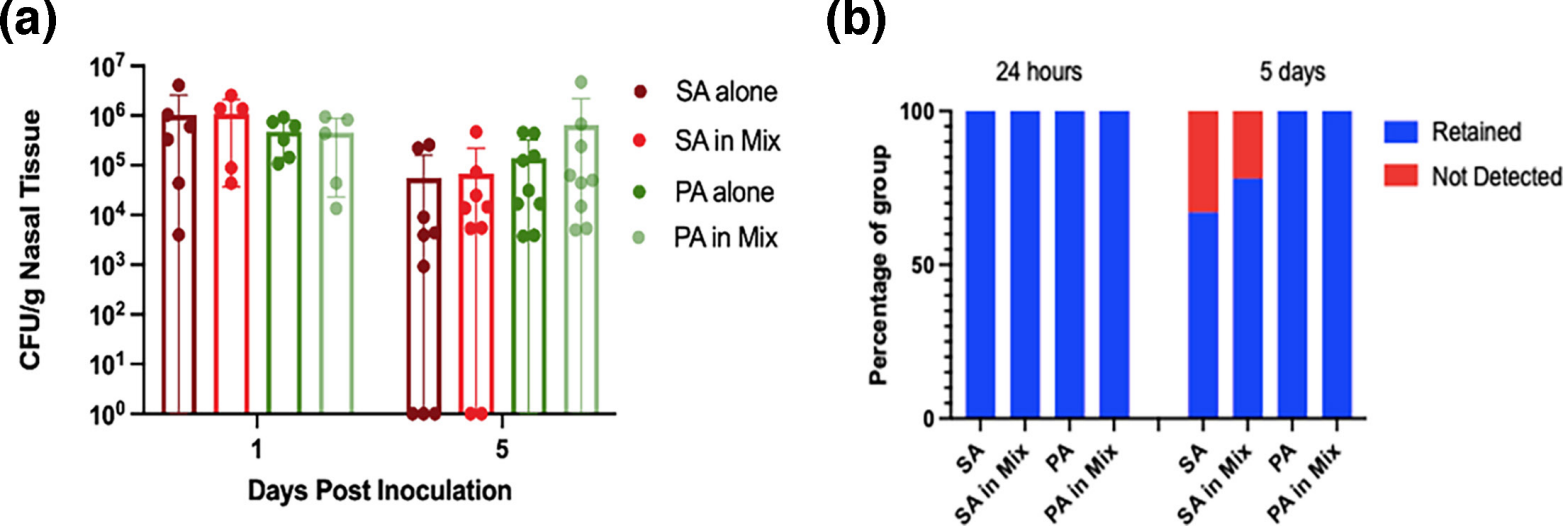
SA & PA sinonasal retention. (a) Sinonasal bacterial load at 24 h and 5 days post-inoculation. Mann−Whitney *U* test to compare c.f.u. for SA vs. SA in Mix and PA vs. PA in Mix was not significant. (b) Rate of retention at 24 h and 5 days post-inoculation. Fisher’s exact test to compare SA vs. SA in Mix and PA vs. PA in Mix was not significant. One day post-inoculation: SA, *n*=6; PA, *n*=6; SA+PA, *n*=5. Five days post-inoculation: n=9 for each group.

Bacterial persistence and host responses were initially assessed in outbred Swiss Webster (SW) mice. The highest bacterial loads for both SA and PA were observed at 24 h post-inoculation (Fig. 1). By day 5, SA persisted at the clinical infection threshold of 10⁵ c.f.u. g^−1^ of tissue, while PA was maintained at slightly higher density [38,39]. To assess persistence, bacterial loads were measured at 24 h and subsequently at 3, 5 and 10 days post-inoculation in a separate experiment (Fig. S1, available in the online Supplementary Material). Although a general decline in bacterial burden was observed over time, both SA and PA remained detectable in more than 50% of mice at day 10, suggesting that this model may be suitable for long-term studies. However, given that host–microbe interactions were the primary focus of this study, bacterial and host responses were assessed at 24 h (acute) and 5 days (subacute), as these time points exhibited high bacterial retention rates.

**Fig. 2.**
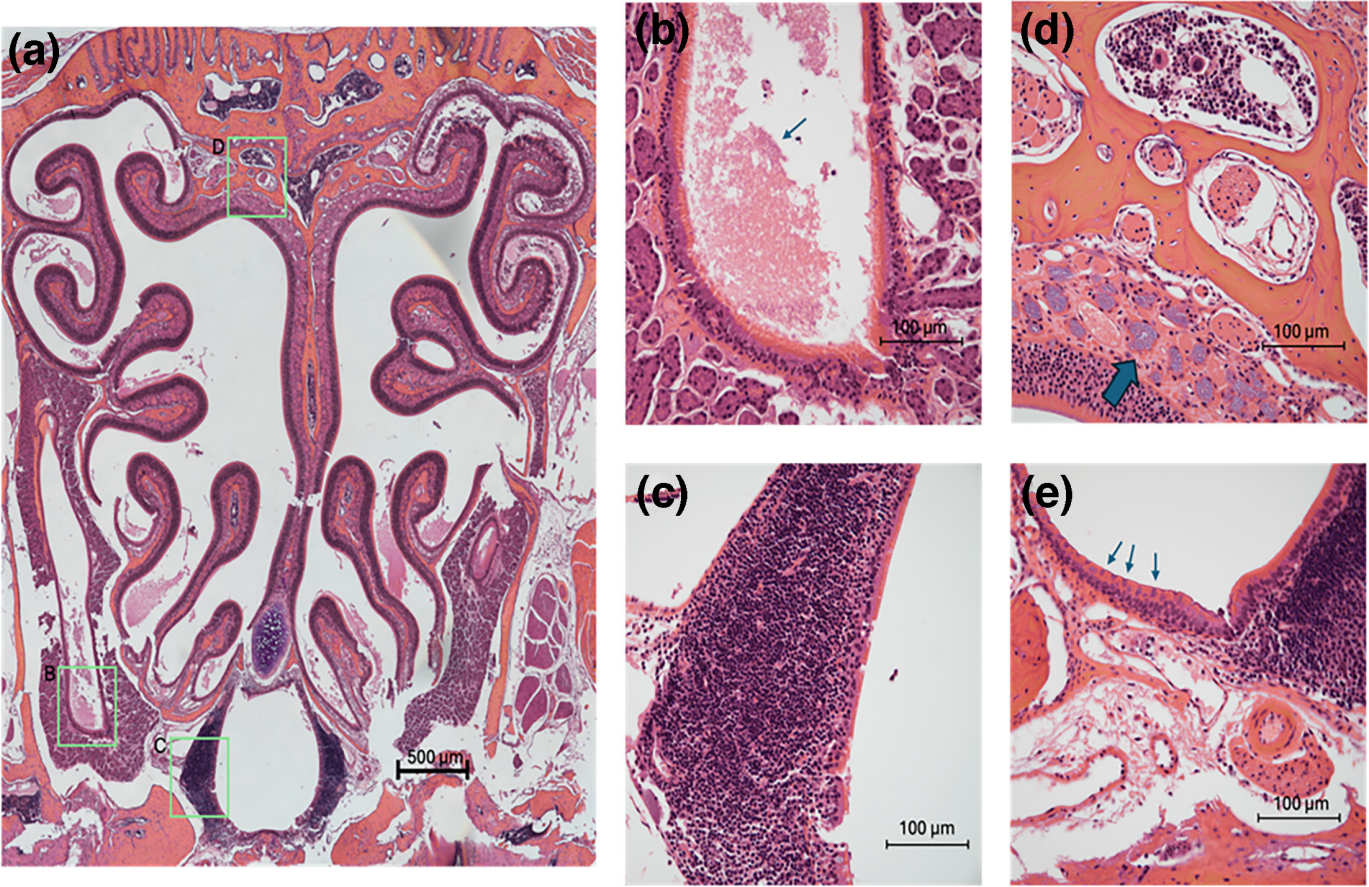
Host responses assessed via histological analysis. Representative images from an SA+PA-inoculated SW mouse at 5 days post-inoculation, stained with haematoxylin and eosin. Panels (b), (c) and (d) are inset. Panel (e) is from a separate mouse in the same experimental group. (a) Composite image of a caudal section of the murine sinonasal cavity at 4× magnification, including turbinates, maxillary sinus, septum and NALT. (b) Exudate (arrow) in the maxillary sinus cavity; 40× magnification. (c) NALT; 40× magnification. (d) The top of the image shows immune cells in the vasculature above the dorsal meatus. The bold arrow shows a Bowman’s gland; 40× magnification. (e) Arrows point to goblet cells near NALT; 40× magnification.

Several histological metrics were quantified to reflect pathological features observed in clinical CRS (Fig. 2). A view of the murine sinonasal cavity at 4× magnification is shown in Fig. 2(a), including turbinates and maxillary sinuses. From this, the epithelial area was measured to assess inflammation and tissue remodelling, key features of CRS pathology that are associated with cytokine signalling pathways, including IL-1*β*, IL-6 and TNF-*α*, as well as the anti-inflammatory IL-10 [21,22, 40,42]. Nasal obstruction, another common CRS symptom, was evaluated by measuring exudate accumulation in nasal passages and sinus cavities as a proxy for nasal discharge (Fig. 2b), which can reflect inflammatory cytokine–driven vascular permeability and mucus production [43,44]. Immune cell recruitment, a characteristic of CRS, was analysed by quantifying those, such as T helper cells, macrophages, neutrophils and eosinophils, in NALT and above the dorsal meatus, where evidence of immune cell trafficking is visible in the vasculature (Fig. 2c, d) [45,48]. These cell populations are commonly associated with cytokine pathways including TNF-*α* and IL-1*β* for macrophage activation, along with KC/GRO for neutrophil recruitment; IL-5 signalling for eosinophilic inflammation and Th2 response; TNF-*α*, INF-*γ*, IL-1*β* and IL-6 for Th1 response; and IL-2 for T-cell proliferation [42,49,51]. Mucus production, a hallmark of CRS, was assessed by quantifying the number and size of Bowman’s glands (Fig. 2d) and goblet cells (Fig. 2e) in the murine sinonasal cavity via histology [52,54]. Bowman’s glands contribute to mucus secretion in the olfactory epithelium, while goblet cells secrete mucus at the epithelial surface of the respiratory epithelium, processes strongly influenced by Th2 cytokines [55,56]. Systemic host responses were evaluated using a multiplex pro-inflammatory cytokine panel to differentiate the host response across the various experimental conditions (see the ‘Methods’ section).

**Fig. 3.**
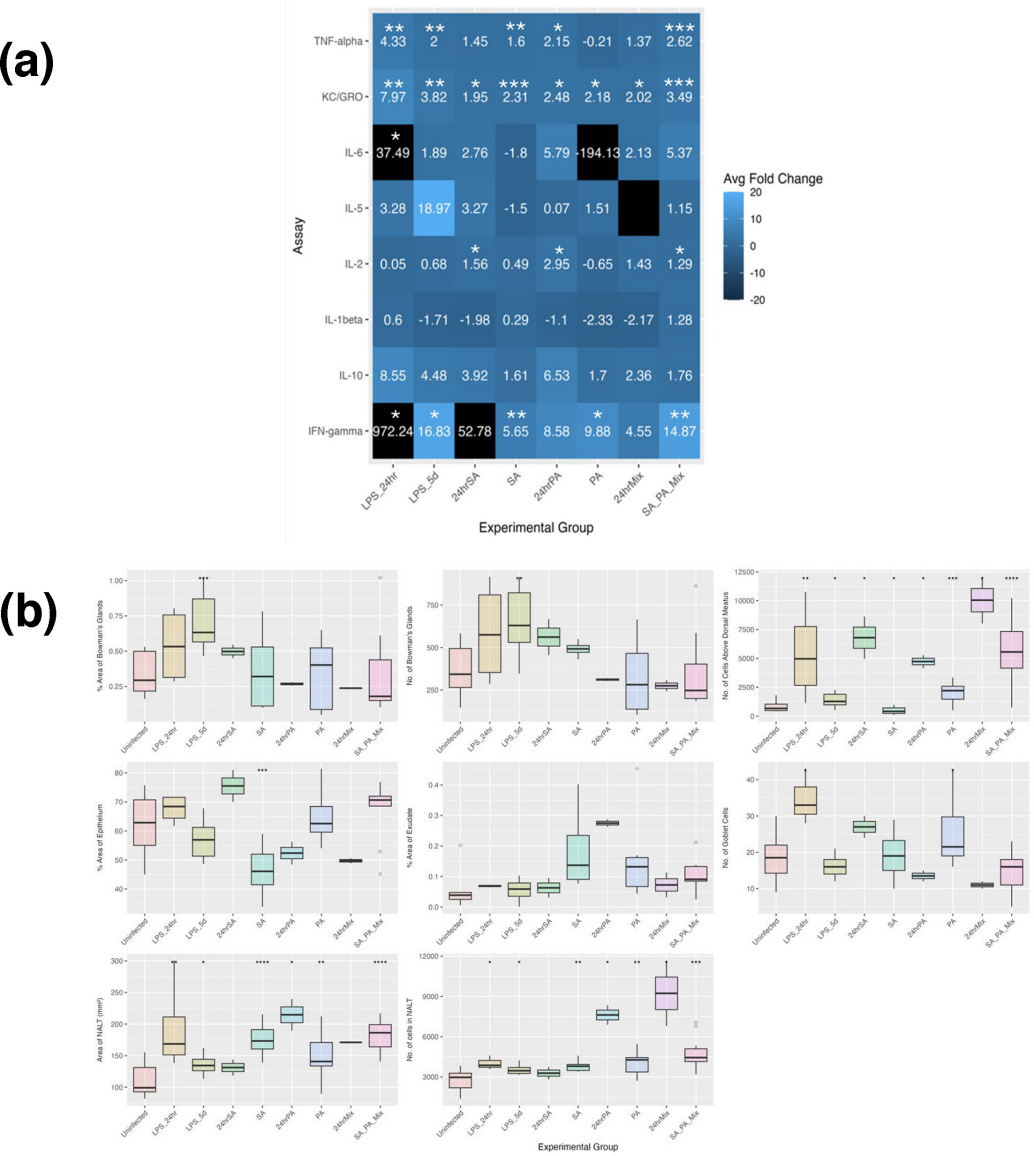
Comparing host responses at 24 h and 5 days post-inoculation. (a) Average adjusted fold change of serum cytokines compared to uninfected mice. Lighter colours indicate increased expression, and dark colours indicate decreased expression. Black indicates expression outside the range of the legend. However, if the fold change does not have a value, it was below the limit of detection of the assay. Stars indicate cytokines with significantly different expression from uninfected mice by the Mann–Whitney *U* test. (b) Histology at 24 h and 5 days post-inoculation. Mann–Whitney *U* test vs. uninfected mice. *P<0.05, **P<0.01, ***P<0.001. Uninfected *n*=8 mice; LPS_24hr, *n*=2; LPS_5d, *n*=2; 24hrSA, *n*=2; SA (5 days post-inoculation), *n*=4; 24hrPA, *n*=2; PA (5 days post-inoculation), *n*=4; 24hrMix, *n*=2; SA_PA_Mix (5 days post-inoculation), *n*=4.

At 24 h post-inoculation (Fig. 3a), TNF-*α* and KC/GRO levels were significantly elevated in both SA- and PA-inoculated groups compared to uninfected controls. Exception for IL-1*β*, all quantified cytokines were elevated, indicating acute inflammation and neutrophil recruitment. Histological analysis corroborated these findings (Fig. 3b), demonstrating a significant increase in immune cells above the dorsal meatus in both SA- and PA-inoculated mice. Additionally, PA-inoculated mice exhibited a significant increase in NALT area and immune cell density within NALT.

At 5 days post-inoculation, IFN-*γ* and KC/GRO remained significantly elevated in both infected groups compared to uninfected controls. IFN-*γ* levels increased relative to the 24 h time point, while IL-2 levels declined. TNF-*α* levels remained significantly elevated in SA-inoculated mice but were not significantly increased in those inoculated with PA, which may suggest a transition of PA to a biofilm phenotype. PA biofilms have been shown to suppress TNF-*α* and IL-2 while promoting IL-10, supporting this interpretation [57]. Collectively, these findings indicate sustained neutrophil and macrophage activation at the 5-day time point.

Histological analysis of site-specific immune responses revealed a significant increase in immune cells within NALT in both SA- and PA-inoculated mice at 5 days post-inoculation (Fig. 3b). In SA-inoculated mice, local inflammation decreased by day 5, as indicated by a reduction in epithelial area and immune cells above the dorsal meatus compared to 24 h post-inoculation. In contrast, PA-inoculated mice exhibited increased epithelial area and goblet cell density between 24 h and 5 days post-inoculation, suggesting persistent inflammation and mucus hypersecretion.

### SA and PA co-inoculation heightens the host response in SW mice

Recent research emphasizes the role of bacterial communities in chronic infections, demonstrating that most persistent infections arise from polymicrobial interactions rather than single-species colonization [58,61]. In CRS, SA and PA are frequently co-isolated, with their co-occurrence associated with worsened disease outcomes and an increased likelihood of revision surgery [62,65]. To investigate the impact of SA–PA interactions in CRS pathogenesis and host responses, co-inoculation experiments were conducted to evaluate colonization dynamics, bacterial retention and host immune activation.

It was hypothesized that co-inoculation of SA (Xen36) and PA (PAO1wLux) would elicit a more robust immune response than single-species inoculation. Additionally, given prior *in vitro* evidence suggesting competitive interactions between SA and PA, it was predicted that PA might reduce SA retention [66,68].

To assess these hypotheses, bacterial retention was evaluated following co-inoculation. At 5 days post-inoculation, SA and PA exhibited slightly higher bacterial loads than in single-species infections, though the differences were not statistically significant (Fig. 1).

The host immune response to co-inoculation exhibited distinct characteristics compared to individual inoculations (Fig. 3). At 24 h post-inoculation, IFN-*γ* expression in co-inoculated mice resembled that of PA-alone infections but was downregulated relative to SA-alone inoculations. In contrast, IL-6 and IL-10 levels mirrored those observed in SA-inoculated mice, with slightly lower expression than in PA-alone infections. Co-inoculation also resulted in significantly increased recruitment of immune cells above the dorsal meatus and in NALT compared to uninfected controls, with levels exceeding those observed in single-species infections. However, mixed inoculation did not induce the same degree of inflammatory response or mucus-producing cell expansion as SA alone, nor did it generate the level of exudate observed with PA infections.

By 5 days post-inoculation, cytokine expression patterns for KC/GRO, IFN-*γ* and TNF-*α* in co-inoculated mice were comparable to those of single-species infections. However, co-inoculation resulted in significantly elevated IL-2 expression and increased levels of IL-6, IL-5, IL-1*β* and IL-10 compared to either species alone, suggesting a unique cytokine response profile. Additionally, epithelial inflammation in co-inoculated mice surpassed that observed in single-species infections. Recruitment of immune cells above the dorsal meatus and NALT cells remained elevated at 5 days post-inoculation, further supporting the distinct immunological impact of SA-PA co-infection.

### Repeated inoculations decrease bacterial retention

Pathogen exposure can alter microbiota composition and induce host changes that create favourable conditions for colonization and infection [69]. Previous studies have examined sinonasal bacterial responses using repeated inoculations over short intervals, such as in Cope *et al*., where three inoculations were administered within 3 days [53]. In contrast, this study investigated the effects of three inoculations spaced 5 days apart in SW mice. It was hypothesized that a longer interval between exposures would lead to an additive effect, increasing bacterial load by day 15. Alternatively, repeated inoculations might enhance bacterial clearance due to immune priming.

**Fig. 4.**
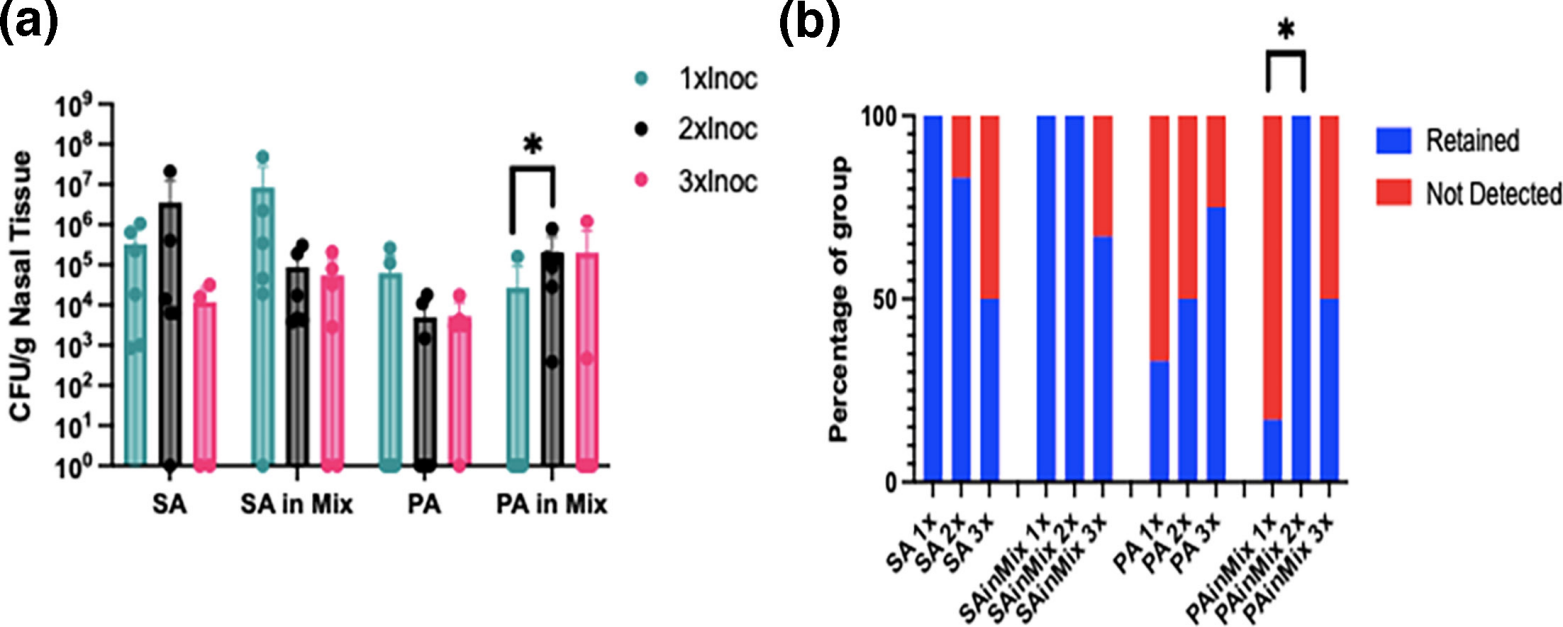
Bacterial load and retention after repeated inoculations. (a) Bacterial load at 5 days post-final inoculation. Mann–Whitney *U* test for PA in Mix 1xInoc vs. PA in Mix 2xInoc, *P=0.0346. (b) Rate of retention of inoculated strains at 5 days post-inoculation. Fisher’s exact tests for retention of PA in Mix 1xInoc vs. PA in Mix 2xInoc, *P=0.0152. SA (Xen36), PA (PAO1wLux). Mix=1:1 SA+PA. *N*=6 mice per group.

As shown in Fig. 4, bacterial load and retention patterns varied with repeated inoculations. In SA-inoculated mice, bacterial load increased after two inoculations but declined following a third exposure. PA-inoculated mice exhibited no significant differences in bacterial load between two and three inoculations, although retention rates increased with each exposure. Despite multiple inoculations, final bacterial loads for both species remained below the clinical infection threshold, suggesting the development of immune memory and enhanced bacterial clearance. In co-inoculated mice, SA load decreased with repeated exposures, whereas PA load and retention increased significantly after two inoculations. Notably, when co-inoculated, both SA and PA maintained bacterial loads near the infection threshold, with retention rates higher than in single-species infections, suggesting potential cooperative interactions. Rather than clear amplification or suppression, the results indicate a moderation of infection.

**Fig. 5.**
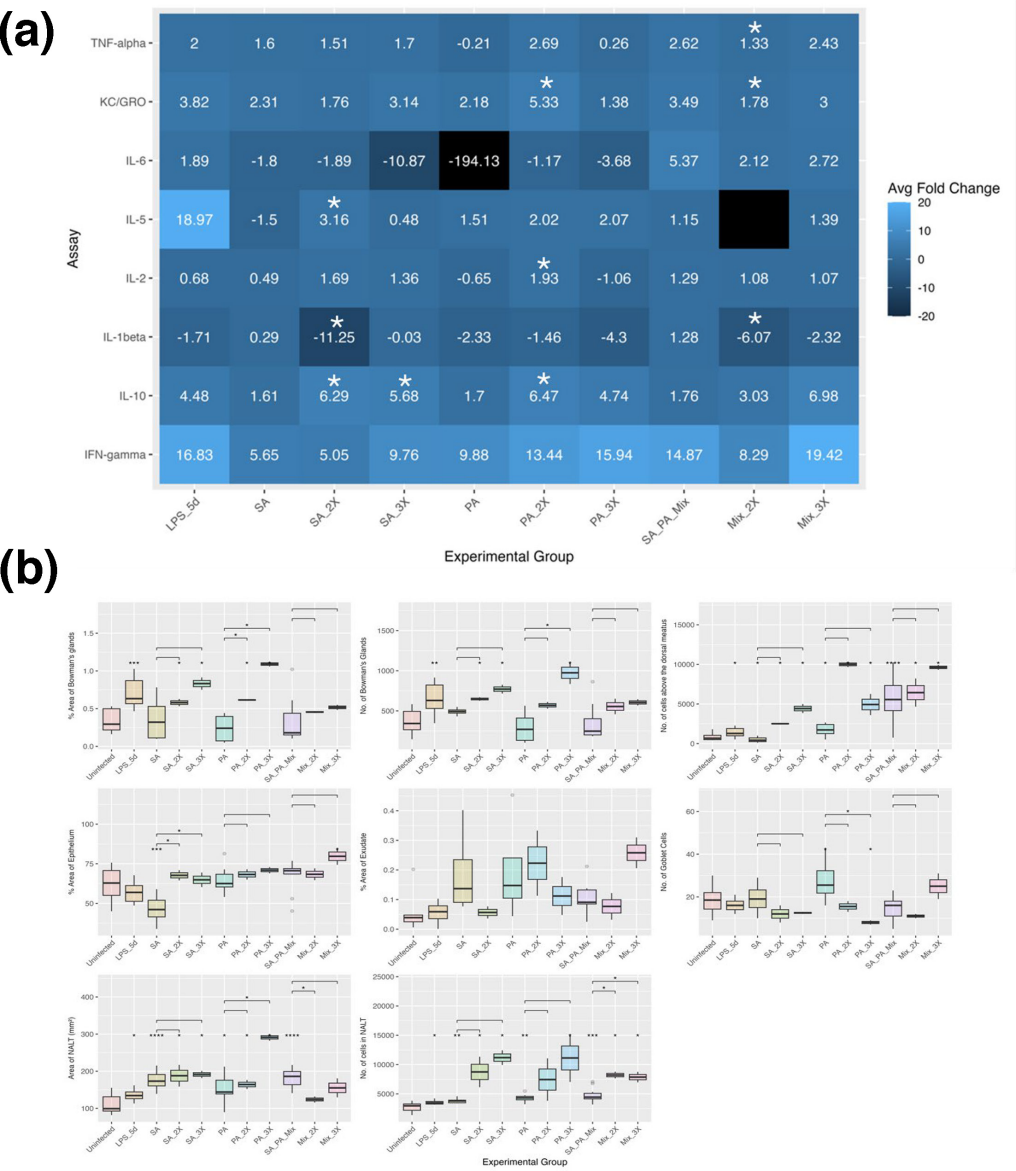
Host response to repeated inoculations. (a) Average adjusted fold change for each cytokine compared to uninfected animals at 5, 10 or 15 days post first inoculation. Lighter colours indicate increased expression, and darker colours indicate decreased expression. Black indicates expression outside the range of the legend. However, if there is no value for fold change, it was below the limit of detection of the assay. Stars indicate pairwise comparisons to single inoculations by the Mann–Whitney *U* test. Uninfected mice, *n*=4; LPS_5d, *n*=3; SA, *n*=6; PA, *n*=5; SA_PA_Mix, *n*=5; repeated inoculations, *n*=2 per group. (b) Histology at 5, 10 or 15 days post first inoculation. Individual stars represent significant changes vs. uninfected mice, and brackets with stars indicate significant changes from pairwise comparisons, both via the Mann–Whitney *U* test. Uninfected, *n*=8 mice; LPS_5d, *n*=2; SA, *n*=4; PA, *n*=4; SA_PA_Mix, *n*=4; repeated inoculations, *n*=2. *P<0.05, **P<0.01, ***P<0.001, ****P<0.0001.

Host response data revealed significant increases in IL-10 following two SA inoculations compared to a single exposure (Fig. 5a). Two SA inoculations also elevated IL-5 levels and reduced IL-1*β*, suggesting a shift away from a Th1-dominated response. In PA-inoculated mice, repeated exposures led to significant increases in IL-2, KC/GRO and IL-10. For histology, repeated PA inoculations significantly increased Bowman’s gland and NALT cell numbers while reducing goblet cell density (Fig. 5b).

Co-inoculations of SA and PA resulted in significant reductions in TNF-*α*, KC/GRO and IL-1*β* after two inoculations, likely reflecting reduced bacterial burden, but these levels rebounded following the third inoculation. According to the histological analyses, co-inoculation also led to a marked increase in NALT cell numbers. Across all groups, IFN-*γ* levels increased with repeated inoculations, though the trend did not reach statistical significance. Overall, the host response data indicate a combination of innate, Th1 and Th2 immune activation.

### Mouse genetic background influences host–microbe interactions

The preceding experiments were conducted using outbred SW mice. To investigate strain-specific responses, inbred Balb/c (Balb) and C57BL/6 (B6) mice were included. Balb mice are considered resistant to chronic PA lung infections, while B6 mice are classified as susceptible [70]. It was hypothesized that susceptibility to lower respiratory tract infection might extend to the upper respiratory tract [71,72]. Therefore, bacterial retention and host responses in B6 and Balb mice were compared to those in SW mice, with the expectation that B6 mice would exhibit higher PA loads and that bacterial retention and immune responses in B6 and Balb mice would be less variable due to their reduced genetic diversity.

**Fig. 6.**
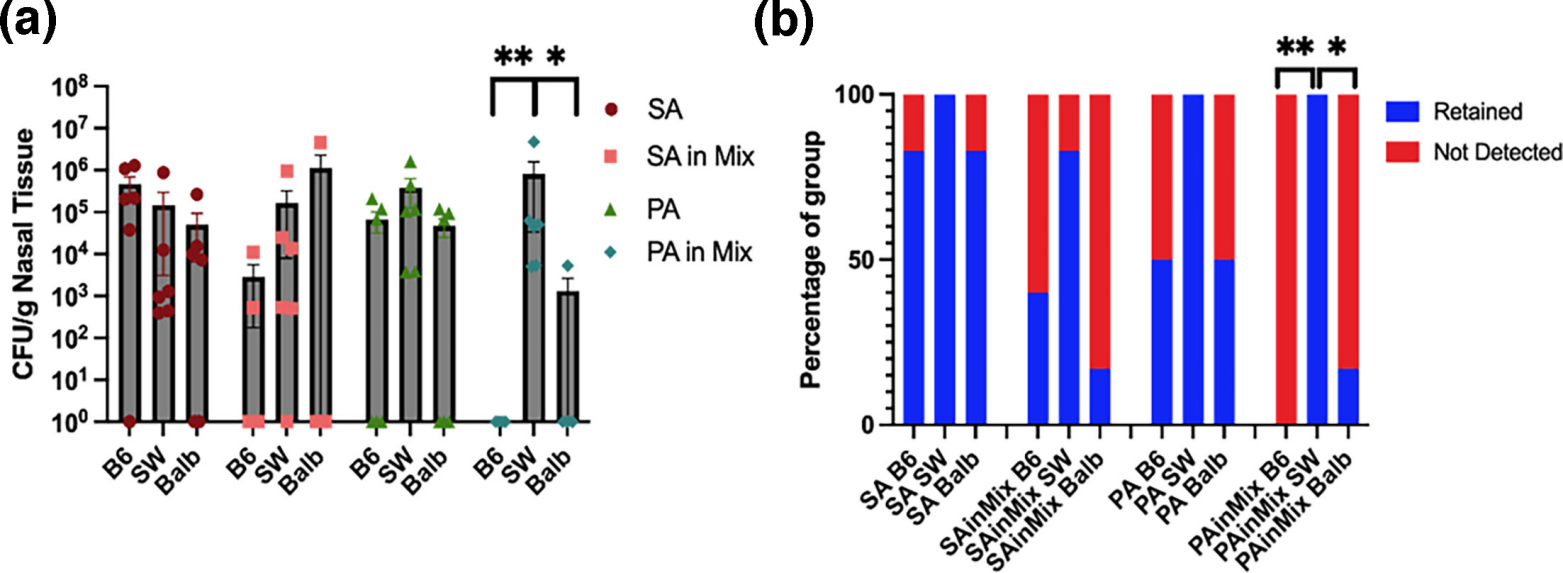
Viable bacteria recovered after inoculation in three mouse strains. (a) Bacterial load at 5 days post-inoculation. Mann–Whitney *U* test for B6 PA vs. SW PA, **P=0.0095; Balb PA vs. SW PA, *P = 0.0190. (b) Rate of retention of inoculated strains at 5 days post-inoculation. Fisher’s exact tests for retention of B6 PA vs. SW PA, **P=0.0022; Balb PA vs. SW PA, *P=0.0152. Mix=1:1 SA+PA. *N*=6 mice per group.

Fig. 6 presents bacterial loads recovered from the sinonasal tissues of the three mouse strains. In single-species inoculations, bacterial load did not significantly differ by strain. Contrary to expectations based on lung infection susceptibility, both Balb and B6 mice harboured PA near the infection threshold at 5 days post-inoculation, with no evidence of dissemination to the lungs. However, in co-inoculations with SA and PA, PA bacterial load and retention were significantly lower in B6 and Balb mice compared to SW mice. These findings suggest that the baseline immune environment influences SA–PA interactions. While differences in sinonasal microbiota may also contribute, no significant differences in bacterial load or morphology were detected in sinonasal tissue from uninfected mice.

**Fig. 7.**
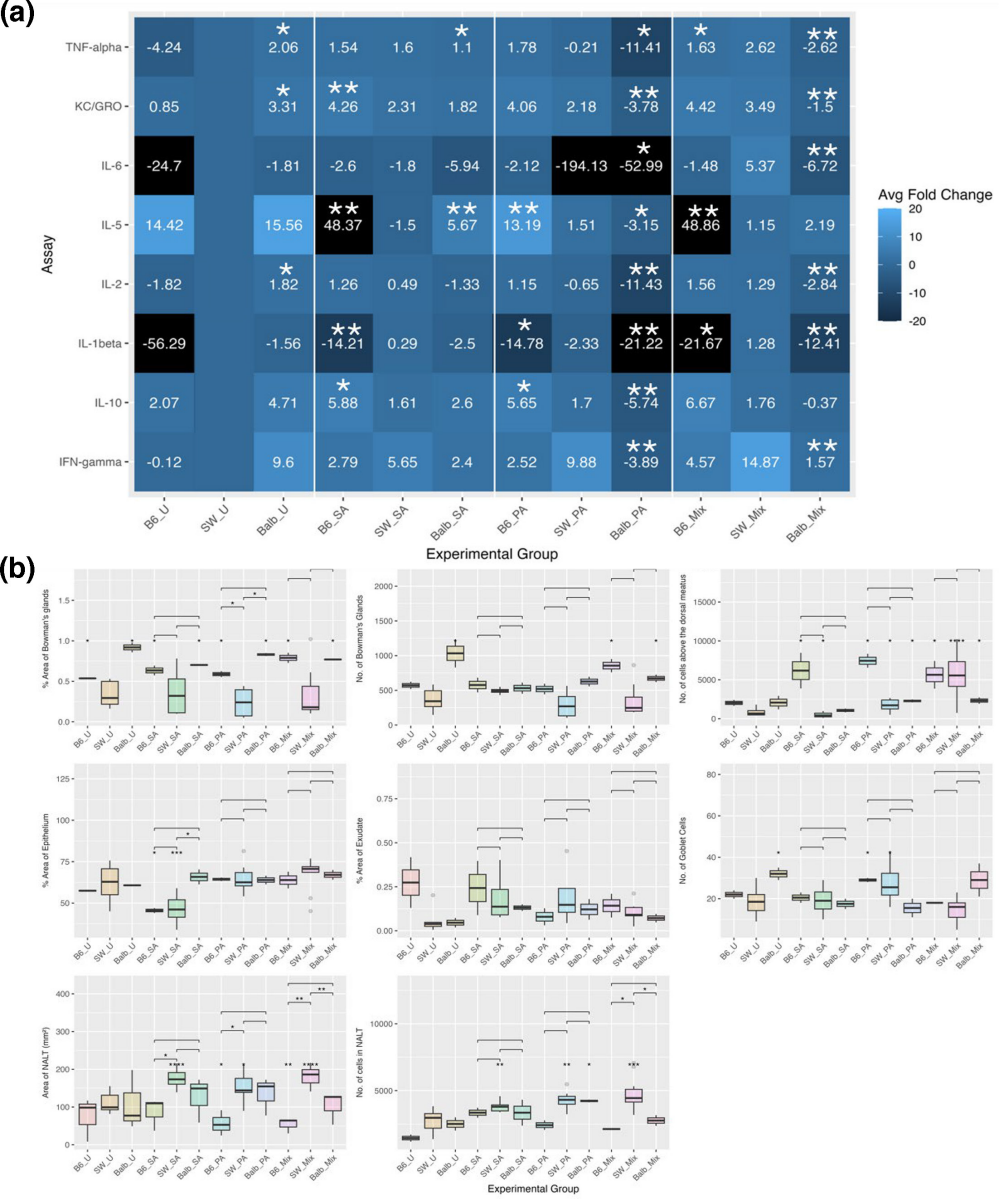
Host responses from B6, Balb and SW after bacterial inoculations. (a) Average adjusted fold change for each cytokine compared to uninfected (SW_U) mice at 5 days post-inoculation. Lighter colours indicate increased expression, and darker colours indicate decreased expression. Black indicates expression outside the range of the legend. However, if there is no value for fold change, it was below the limit of detection of the assay. Stars indicate pairwise comparisons to inoculations in SW mice by the Mann–Whitney *U* test. Mix=1:1 SA+PA. Uninfected (U). SW_U, *n*=4; SW_SA, *n*=6; SW_PA, *n*=5; SW_Mix, *n*=5; B6 and Balb, *n*=2 per group. (b) Histology at 5 days post-inoculation. Individual stars represent significant changes vs. uninfected (SW_U) mice, and brackets with stars indicate significant changes from pairwise comparisons, both via the Mann–Whitney *U* test. Comparisons within the mouse strain for B6 and Balb mice were not significant. SW_U, *n*=8; SW_SA, *n*=4; SW_PA, *n*=4; SW_Mix, *n*=4; B6 and Balb, *n*=2 mice per group. *P<0.05, **P<0.01, ***P<0.001, ****P<0.0001.

Baseline cytokine expression varied across strains, with uninfected Balb mice exhibiting significantly higher TNF-*α*, KC/GRO and IL-2 levels than both B6 and SW mice (Fig. 7a). Following bacterial inoculation, cytokine levels in Balb mice generally decreased compared to uninfected controls. This resulted in significantly altered expression of eight cytokines in PA-inoculated Balb mice compared to SW mice and six cytokines in Balb mice co-inoculated with SA+PA. However, no cytokines were significantly different compared to uninfected Balb mice, suggesting substantial variability within this group.

Histological analysis supported these observations (Fig. 7b). Uninfected Balb mice exhibited significantly larger and more numerous Bowman’s glands and goblet cells, indicating enhanced mucociliary clearance as a primary defence mechanism. Despite reduced cytokine levels, Balb mice exhibited significantly increased epithelial inflammation following SA inoculation. Additionally, co-inoculation with SA and PA resulted in reduced immune cell recruitment to both NALT and the area above the dorsal meatus compared to SW mice and single-species infections within the Balb strain.

In contrast, bacterial inoculation in B6 mice led to increased cytokine expression compared to uninfected controls. Exception for IL-5, which was lower in PA-inoculated B6 mice, cytokine responses remained consistent across all bacterial inoculations. This contrasts with the more variable responses observed in Balb and SW mice, where cytokine expression was influenced by bacterial species and co-inoculation. Additionally, B6 mice exhibited increased immune cell recruitment above the dorsal meatus across all bacterial inoculations compared to SW and Balb mice but demonstrated a significant reduction in NALT across all conditions.

### SA and PA elicit disparate outcomes in severe combined immunodeficient mice

B6.Cg-*Prkdc^scid^*/SzJ [severe combined immunodeficient (SCID)] mice, which lack functional T and B cells but retain normal antigen-presenting cells, myeloid cells and natural killer cells, were selected to investigate the role of innate immunity in murine sinonasal bacterial infection [73]. It was hypothesized that SCID mice would exhibit impaired bacterial clearance due to the absence of adaptive immunity, leading to higher bacterial retention [74]. Additionally, it was predicted that SCID mice would have diminished cytokine responses, particularly in IL-2, IL-10, IFN-*γ*, IL-6 and TNF-*α*, due to the lack of adaptive contribution, compared to immunocompetent B6 controls.

**Fig. 8.**
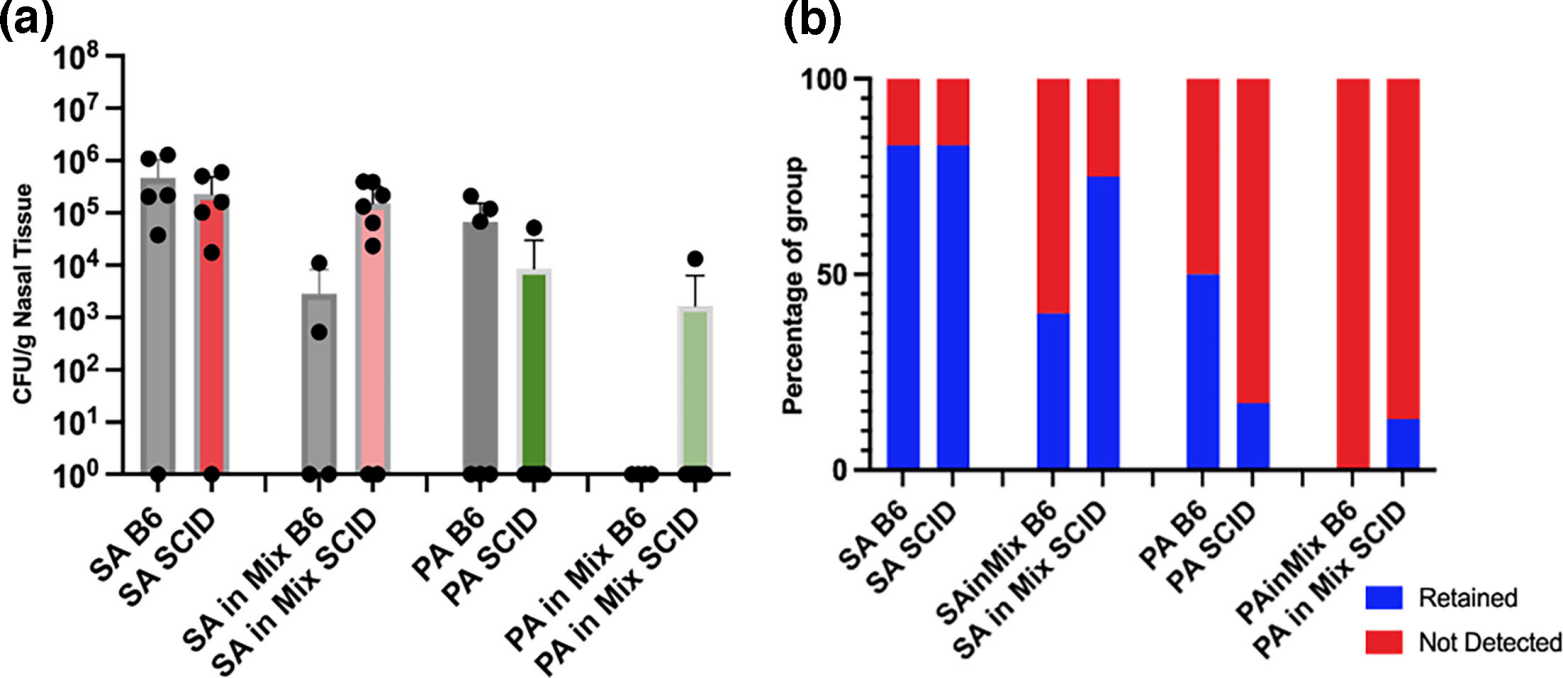
Bacterial viability following inoculation in SCID animals. (a) Bacterial load in sinonasal tissue 5 days post-inoculation. Pairwise comparisons of B6 vs. SCID by inoculation were not significant via the Mann–Whitney *U* test. (b) Rate of retention of inoculated strains at 5 days post-inoculation. Pairwise comparisons of B6 vs. SCID by inoculation were not significant via Fisher’s exact test. Mix=1:1 SA+PA. B6, *n*=6 per group. SA SCID, *n*=6; PA SCID, *n*=6; SA+PA SCID, *n*=8.

Strikingly, at 5 days post-inoculation, PA was cleared in all but one of the SCID mice following single-species infection, whereas SA persisted at levels comparable to B6 controls (Fig. 8). This suggests that SA may evade innate immune clearance mechanisms more effectively than PA. In co-inoculated SCID mice, PA was detected in only one mouse, while SA persisted, a pattern that mirrored single-species infections. This likely reflects differential innate immune responses to Gram-positive vs. Gram-negative pathogens rather than direct bacterial antagonism [75].

**Fig. 9.**
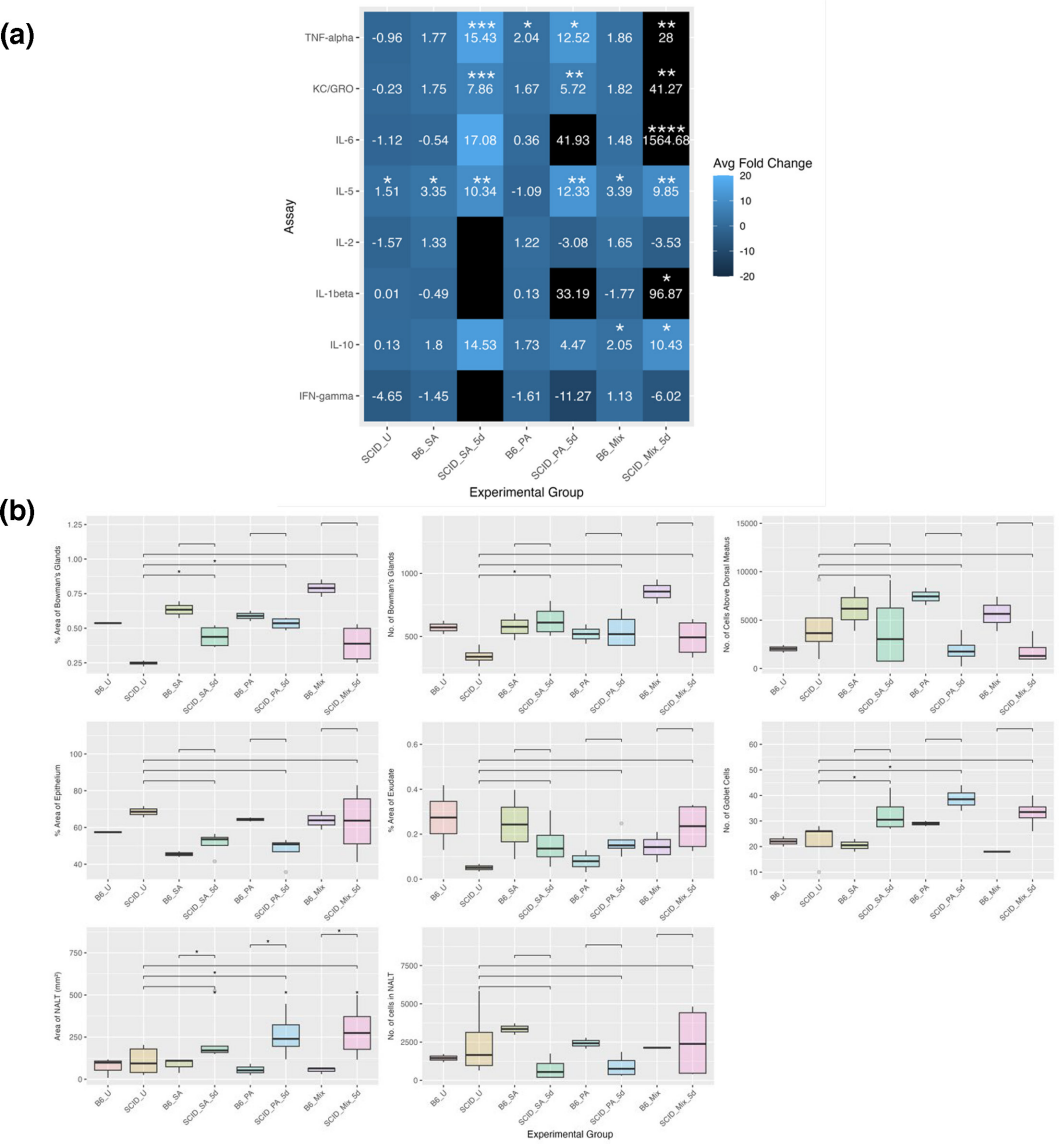
Host responses in SCID mice following sinonasal bacterial inoculation. (a) Average adjusted fold change for each cytokine compared to uninfected (B6_U) animals at 5 days post-inoculation. Lighter colours indicate increased expression, and darker colours indicate decreased expression. Black indicates expression outside the range of the legend. However, if there is no value for fold change, it was below the limit of detection of the assay. Stars indicate pairwise comparisons to uninfected B6 animals by the Mann–Whitney *U* test. (b) Histology at 5 days post-inoculation. Individual stars indicate significant differences vs. uninfected (B6_U) mice by the Mann–Whitney *U* test. Brackets indicate pairwise comparisons with uninfected SCID mice (SCID_U) and B6 inoculations. *N*=4 for all SCID groups. *P<0.05, **P<0.01, ***P<0.001, ****P<0.0001.

As shown in Fig. 9, uninfected SCID mice exhibited significantly elevated IL-5 levels compared to B6 mice, along with an increased goblet cell count across all SCID groups. However, SCID mice had fewer and smaller Bowman’s glands, and immune cell numbers in NALT were significantly reduced relative to B6 controls, consistent with their immunodeficient phenotype.

The cytokine response to SA inoculation in SCID mice resulted in upregulated expression compared to that in B6 mice. Notably, SA-inoculated SCID mice were the only group to exhibit a significantly increased number of Bowman’s glands relative to uninfected SCID mice. KC-GRO and TNF-*α* and IL-5 levels in SA-inoculated SCID mice were significantly higher than in B6 controls, indicating strong macrophage activation and a pronounced neutrophilic inflammatory response along with eosinophil activation, countering the hypothesis that SA colonization in these mice would be treated as commensal.

In contrast, PA-inoculated SCID mice exhibited reduced IFN-*γ* and IL-2 levels compared to B6 controls, likely reflecting both the anticipated reduction in cytokine production and resolution of the immune response, as PA was only detected in one of the SCID mice at 5 days post-inoculation. In co-inoculated SCID mice, IFN-*γ* levels remained lower than in B6 controls, with reduced immune cell recruitment above the dorsal meatus, mirroring the PA-only response. However, IL-1*β* was significantly elevated, mirroring the macrophage activation of the SA response, and dramatically increased IL-6 levels, with some increase in epithelial area, suggesting a robust inflammatory response.

## Discussion

CRS presents significant challenges in clinical management due to the heterogeneity of its pathological endotypes and the persistent presence of bacteria and biofilm [76,77]. Many patients require prolonged treatment, prompting efforts to better understand CRS pathogenesis and identify therapeutic targets [8,78, 79]. While various animal models exist, they often fail to capture microbial contributions to disease progression [80,81]. This study aimed to establish an accessible murine sinonasal infection model capable of quantifying bacterial persistence and host immune responses, providing a more precise tool for studying CRS pathogenesis.

Inoculation of CRS-associated bacterial species successfully established sinonasal colonization and induced histological and cytokine responses consistent with clinical CRS. Quantifying bacterial load over time (24 h to 10 days) provided greater resolution than many previous models, which often rely on presence/absence detection or relative abundance at early time points [53,82]. Bacterial loads remained stable near 10⁵ c.f.u. g^−1^ of tissue from 24 h to 5 days, consistent with the clinical infection threshold for wounds, urinary and respiratory tract infections [38,39, 83,85]. This level of persistence, although lower than in other SA and PA infection models, may prevent bacterial dissemination to the lower airways and also may alter bacterial interactions and host responses compared to the higher bacterial density models [86,87]. This moderate load also reflects clinical observations of bacterial burden in CRS, further emphasizing the translatability of the model [77,88].

Based on the notorious biofilm-forming capacity of SA and PA individually and in combination, we expect that a biofilm was formed in the murine sinonasal cavity during these experiments. Cytokine patterns, such as the regulation of TNF-*α* and IL-2 in favour of IL-10, support this hypothesis. However, the bacterial burden and anatomy of the murine sinonasal epithelial surface complicated microscopic evaluation of the inoculated strains, which is one way to confirm the presence of biofilm in the model. *Ex vivo* antibiotic tolerance could also be used in future CRS models as a proxy for biofilm formation.

Co-inoculation with SA and PA resulted in an immune response distinct from single-species infections, characterized by increased macrophage activation, neutrophil recruitment and T-cell signalling. These changes were supported by histological evidence of increased immune cell populations in NALT and above the dorsal meatus. The heightened inflammatory response in mixed infections suggests that bacterial interactions contribute to CRS pathogenesis and these contributions cannot be fully captured using single-species inoculation models.

SA and PA are both well equipped with numerous virulence factors that may influence host–microbe interactions within this model [80]. For example, alpha and beta toxins from SA impair cilia and activate host inflammation, while pyocyanin from PA can skew T helper responses toward Th2 [89,90]. Furthermore, when SA and PA are co-inoculated, alginate production by PA can result in altered virulence expression, leading to heightened tolerance to antibiotics by SA [91,92]. Thus, antagonistic, cooperative and neutral interactions may play a role in the outcomes observed in these experiments.

While simultaneous exposure and nasal colonization by SA and PA together may be relatively uncommon, one clinical cohort reported that the organisms co-occurred in ~17% of sinusitis cases [65]. Future work using staggered inoculation could clarify how early establishment of SA alters interactions with PA, or vice versa, which may have a downstream impact on host responses.

Though SA and PA are commonly identified in CRS, numerous other microbes play a role, including *Moraxella*, *Haemophilus*, *Streptococcus*, *Peptostreptococcus* and *Prevotella*. Here, we used the two pathogens to establish a controlled framework for investigating pathogen-specific host responses within the murine sinonasal cavity. Ongoing work in our lab is characterizing these responses in multi-species inoculations. However, modern microbiological disease research necessitates the incorporation of community-level investigations, which are currently underdeveloped and remain complex to implement and evaluate *in vivo*. This must be the focus of future work.

Repeated inoculations suggest that adaptive immunity may play a role in the management of bacterial burden for SA or PA alone. However, neither species was cleared, and the increased density of bacteria remaining in the sinonasal cavity after three SA+PA inoculations mimics the lack of bacterial clearance observed in CRS patients. Extended time points such as those herein (5, 10 and 15 days) are essential for modelling and investigating the chronicity of CRS.

In these experiments, the host immune response to sinonasal infection varied by genetic background. Human studies indicate that CRS may be associated with genetic variations in Th2 cytokines, innate immune pathways, epithelial barrier, oxidative stress and vascular permeability [93,96]. When autoimmune phenotypes were compared to CRS, genetic correlations were identified with allergic rhinitis, asthma, rheumatoid arthritis, hypothyroidism and type 1 diabetes [97]. Given that human CRS is genetically heterogeneous and polygenic, outbred SW mice were utilized to approximate the spread of susceptibility alleles and variable penetrance seen in patients. Balb are considered classically Th2-biassed and tend to develop more intense allergic/eosinophilic airway and sinonasal inflammation than B6 [98]. In fact, B6 mice are reported to have skewed Th1 responses in the airway at baseline, though they have the potential for subsequently exaggerated Th2 responses during incidences of airway inflammation [99]. Utilizing these inbred strains models the spectrum of innate immune activation observed in CRS. Finally, without functional T helper or B cell response, SCID mice maintain an intact innate immune response [100]. Therefore, they are useful in elucidating intrinsic epithelial contributions to CRS compared to adaptive-immune genetic contributions in experiments with B6 controls.

To this end, Balb mice exhibited heightened baseline cytokine levels but downregulated their response following infection, suggesting a homeostatic immune modulation strategy. In contrast, B6 mice exhibited robust neutrophil and macrophage recruitment, consistent with prior findings of increased innate immune activation in B6 syngeneic transplant and lung infection models [101,102]. These results highlight the importance of host factors in shaping CRS-associated inflammation.

This study represents the first description of sinonasal infection in SCID mice to our knowledge. Uninfected SCID mice exhibited elevated baseline IL-5 and increased goblet cell numbers, potentially as a compensatory mechanism. Despite the absence of adaptive immunity, SCID mice effectively cleared PA but retained SA in both single and dual-species inoculations. This suggests that innate immunity alone is sufficient for PA clearance but not for SA, possibly due to SA’s ability to evade neutrophil-mediated clearance [103]. We are not the first to demonstrate that SCID mice can clear PA infection, as Fruh *et al*. demonstrated that SCID mice survive systemic PA exposure at 1.4×10^6^ c.f.u. ml^−1^ for up to 3 days post-inoculation [104]. This is likely a non-specific response to bacterial lipopolysaccharide, as additionally evidenced by response to systemic *Salmonella typhimurium* infection and exogenous LPS in SCID mice [105]. During this response, Fruh *et al*. demonstrated that bacterial LPS activates macrophages, inducing the production of TNF-*α*, which then activates natural killer cells to release IFN-*γ*, further activating macrophages and stimulating phagocytosis [104]. We show that the same response is true for local sinonasal infection with PA in SCID mice. This suggests that regulation of the innate immune response by the adaptive immune response may be counterproductive in the immune management of Gram-negative pathogenic bacteria in CRS, but may be required for pathogens like SA, which can evade the same innate immune surveillance. Unlike respiratory xenografts infected with bacteria in immune-compromised animals [106,107], SCID mice in this study also retained intact cilia and increased goblet cells, elements of effective mucociliary clearance, which may facilitate PA elimination.

These findings reflect the complexity of CRS pathogenesis, demonstrating that bacterial interactions, host genetic background and immune competence all contribute to disease progression. The SCID model provides a valuable tool for studying innate immune evasion by SA, while the sinonasal infection model as a whole offers a microbiologically relevant approach for investigating CRS-associated host–microbe interactions. Future studies should explore additional CRS-relevant bacterial species and assess biofilm formation to further refine this model. Overall, this study enhances the understanding of CRS-associated inflammation and supports the use of a murine sinonasal infection model as a practical tool for investigating bacterial contributions to CRS and potential therapeutic interventions.

## Supplementary material

10.1099/jmm.0.002152 Uncited Fig. S1.

## Abbreviations

CRS: chronic rhinosinusitis
FOV: fields of view
LPS: lipopolysaccharide
NALT: nasal-associated lymphoid tissue
PA: *Pseudomonas aeruginosa*
SA: *Staphylococcus aureus*
SCID: severe combined immunodeficient
SW: Swiss Webster
