# Supplementary material for: A murine sinonasal infection model recapitulates key features of clinical chronic rhinosinusitis
Source: J Med Microbiol. 2026 Apr 8;75(4):002152. doi: 10.1099/jmm.0.002152 (PMC13061426; doi:10.1099/jmm.0.002152)
Supplement: Uncited Fig. S1. [file jmm-75-02152-s001.pdf]

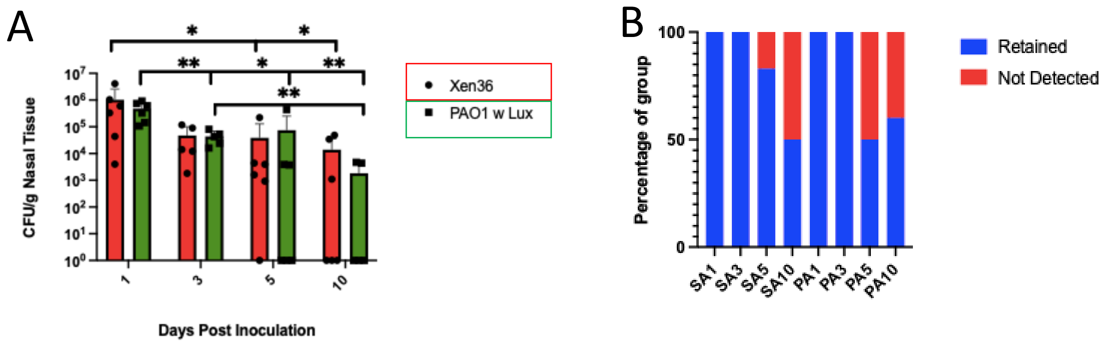

Supplemental Figure 1 Burden and retention rate of inoculated strains over 10 days

A) Bacterial load at days 1, 3, 5 & 10 post inoculation. Kruskal-Wallis Test followed by Mann-Whitney U test *$p < 0.05$, **$p < 0.01$, ***$p < 0.001$. **B)** Rate of retention of inoculated strains by days post inoculation. Fisher's Exact Test was not significant. Days 1, 5, & 10 post inoculation, n=6 mice per species; Day 3 post inoculation, n=5 mice per species.
